# Supplementary material for: Active site specificity profiling datasets of matrix metalloproteinases (MMPs) 1, 2, 3, 7, 8, 9, 12, 13 and 14
Source: Data Brief. 2016 Feb 22;7:299–310. doi: 10.1016/j.dib.2016.02.036 (PMC4777984; doi:10.1016/j.dib.2016.02.036)
Supplement: Supplementary file 10 — Supplementary material [file mmc10.zip › WebPICS_hMMP7_G_1%/P2.html]

 

PICS results


|  |  |
| --- | --- |
| **P2\_A**  22 in 163 sites   13.5 %    effects > 10 perc. pnts.  (vice-versa in brackets)  P3prime\_K: 16.5 (14.5) |  |
| **P2\_C**  4 in 163 sites   2.5 %    effects > 10 perc. pnts.  (vice-versa in brackets)  P3prime\_C: 23.2 (30.8) |  |
| **P2\_H**  5 in 163 sites   3.1 %    effects > 10 perc. pnts.  (vice-versa in brackets)  P1\_C: 17.5 (21.9) |  |
| **P2\_K**  14 in 163 sites   8.6 %    effects > 10 perc. pnts.  (vice-versa in brackets)  P3\_P: 11.6 (10.2)   P2prime\_I: 11.6 (10.2)   P2prime\_R: 12.2 (11.4)   P3prime\_S: 14.7 (18.7)   P3prime\_V: 13.4 (14.5) |  |
| **P2\_L**  18 in 163 sites   11.0 %    effects > 10 perc. pnts.  (vice-versa in brackets)  P1\_P: 21.7 (39.0)   P1\_R: 11.2 (11.2) |  |
| **P2\_M**  5 in 163 sites   3.1 %    effects > 10 perc. pnts.  (vice-versa in brackets)  P1\_H: 16.9 (16.9)   P1prime\_M: 35.1 (21.9) |  |
| **P2\_N**  13 in 163 sites   8.0 %    effects > 10 perc. pnts.  (vice-versa in brackets)  P3\_I: 15.7 (17.0)   P1\_G: 23.4 (25.3)   P2prime\_R: 13.9 (12.0)   P2prime\_T: 13.3 (10.8) |  |
| **P2\_Q**  11 in 163 sites   6.7 %    effects > 10 perc. pnts.  (vice-versa in brackets)  P1\_S: 27.8 (21.9)   P2prime\_L: 24.1 (13.3)   P3prime\_A: 28.3 (11.2) |  |
| **P2\_S**  13 in 163 sites   8.0 %    effects > 10 perc. pnts.  (vice-versa in brackets)  P1\_Q: 21.6 (18.7)   P2prime\_Q: 16.4 (19.3) |  |
